# Supplementary material for: A Critical View of the Application of the APEX Software (Aqueous Photochemistry of Environmentally-Occurring Xenobiotics) to Predict Photoreaction Kinetics in Surface Freshwaters
Source: Molecules. 2019 Dec 18;25(1):9. doi: 10.3390/molecules25010009 (PMC7017383; doi:10.3390/molecules25010009)
Supplement: Supplementary file 1 [file molecules-25-00009-s001.zip › 3.2.4_gcc-4.4.0.zip!/3.2.4_gcc-4.4.0/3.2.4_gcc-4.4.0/doc/octave/html/interpreter/Command-Line-Options.html]

Command Line Options - Untitled


Next: Startup Files,
Up: Invoking Octave from the Command Line


---

#### 2.1.1 Command Line Options

Here is a complete list of the command line options that Octave
accepts.

`--debug` `-d`: Enter parser debugging mode. Using this option will cause Octave's parser to print a lot of information about the commands it reads, and is probably only useful if you are actually trying to debug the parser. `--doc-cache-file` filename: Specify the name of the doc cache file to use. The value of filename specified on the command line will override any value of `OCTAVE_DOC_CACHE_FILE` found in the environment, but not any commands in the system or user startup files that use the `doc_cache_file` function. `--echo-commands` `-x`: Echo commands as they are executed. `--eval` code: Evaluate code and exit when finished unless `--persist` is also specified. `--exec-path` path: Specify the path to search for programs to run. The value of path specified on the command line will override any value of `OCTAVE_EXEC_PATH` found in the environment, but not any commands in the system or user startup files that set the built-in variable `EXEC_PATH`. `--help` `-h` `-?`: Print short help message and exit. `--image-path` path: Add path to the head of the search path for images. The value of path specified on the command line will override any value of `OCTAVE_IMAGE_PATH` found in the environment, but not any commands in the system or user startup files that set the built-in variable `IMAGE_PATH`. `--info-file` filename: Specify the name of the info file to use. The value of filename specified on the command line will override any value of `OCTAVE_INFO_FILE` found in the environment, but not any commands in the system or user startup files that use the `info_file` function. `--info-program` program: Specify the name of the info program to use. The value of program specified on the command line will override any value of `OCTAVE_INFO_PROGRAM` found in the environment, but not any commands in the system or user startup files that use the `info_program` function. `--interactive` `-i`: Force interactive behavior. This can be useful for running Octave via a remote shell command or inside an Emacs shell buffer. For another way to run Octave within Emacs, see Emacs Octave Support. `--line-editing`: Force readline use for command-line editing. `--no-history` `-H`: Disable recording of command-line history. `--no-init-file`: Don't read the initialization files ~/.octaverc and .octaverc. `--no-init-path`: Don't initialize the search path for function files to include default locations. `--no-line-editing`: Disable command-line editing. `--no-site-file`: Don't read the site-wide octaverc initialization files. `--norc` `-f`: Don't read any of the system or user initialization files at startup. This is equivalent to using both of the options `--no-init-file` and `--no-site-file`. `--path` path `-p` path: Add path to the head of the search path for function files. The value of path specified on the command line will override any value of `OCTAVE_PATH` found in the environment, but not any commands in the system or user startup files that set the internal load path through one of the path functions. `--persist`: Go to interactive mode after `--eval` or reading from a file named on the command line. `--silent` `--quiet` `-q`: Don't print the usual greeting and version message at startup. `--traditional` `--braindead`: For compatibility with matlab, set initial values for user preferences to the following values ``` PS1 = ">> " PS2 = "" beep_on_error = true confirm_recursive_rmdir = false crash_dumps_octave_core = false default_save_options = "-mat-binary" fixed_point_format = true history_timestamp_format_string = "%%-- %D %I:%M %p --%%" page_screen_output = false print_empty_dimensions = false ``` and disable the following warnings ``` Octave:fopen-file-in-path Octave:function-name-clash Octave:load-file-in-path ``` `--verbose` `-V`: Turn on verbose output. `--version` `-v`: Print the program version number and exit. file: Execute commands from file. Exit when done unless `--persist` is also specified.

Octave also includes several functions which return information
about the command line, including the number of arguments and all of the
options.

— Built-in Function: **argv** ()  
> Return the command line arguments passed to Octave. For example,
> if you invoked Octave using the command
>
> ```
>           octave --no-line-editing --silent
> ```
>
> `argv` would return a cell array of strings with the elements
> `--no-line-editing` and `--silent`.
>
> If you write an executable Octave script, `argv` will return the
> list of arguments passed to the script. See Executable Octave Programs,
> for an example of how to create an executable Octave script.

— Built-in Function: **program\_name** ()  
> Return the last component of the value returned by
> `program_invocation_name`.
>
>
>
> **See also:** program\_invocation\_name.

— Built-in Function: program\_invocation\_name **(**)  
> Return the name that was typed at the shell prompt to run Octave.
>
> If executing a script from the command line (e.g., `octave foo.m`)
> or using an executable Octave script, the program name is set to the
> name of the script. See Executable Octave Programs, for an example of
> how to create an executable Octave script.
>
>
>
> **See also:** program\_name.

Here is an example of using these functions to reproduce the command
line which invoked Octave.

```
     printf ("%s", program_name ());
     arg_list = argv ();
     for i = 1:nargin
       printf (" %s", arg_list{i});
     endfor
     printf ("\n");
```

See Indexing Cell Arrays, for an explanation of how to retrieve objects
from cell arrays, and Defining Functions, for information about the
variable `nargin`.
